# Study Burnout and Engagement During COVID-19 Among University Students: The Role of Demands, Resources, and Psychological Needs

**DOI:** 10.1007/s10902-022-00518-1

**Published:** 2022-04-01

**Authors:** Katariina Salmela-Aro, Katja Upadyaya, Inka Ronkainen, Lauri Hietajärvi

**Affiliations:** Faculty of Educational Sciences, University of Helsinki, Siltavuorenpenger 5, Helsinki, Finland

**Keywords:** COVID-19, Higher education, Self-determination theory, Demands-resources, Burnout, Engagement

## Abstract

The COVID-19 pandemic forced most universities to switch from in-person to remote teaching from May 2020 to May 2021. This period covered three semesters of studies, and due to these changes students experienced fundamental changes in their learning. The present research was carried out 3 times during the pandemic (e.g., May 2020, December 2020, and April 2021) to investigate study engagement and burnout, and their associations with various demands, resources, and psychological needs among university students. Self-reports were collected from 1501, 1526, and 1685 university students in Helsinki. The results showed that study burnout increased across the time points, being the highest in April 2021, whereas study engagement was the lowest in December 2020. Further, at the beginning of the pandemic the explanatory power of study-related demands and resources on study burnout and engagement was stronger, whereas in April 2021 the role of psychological needs increased. These results inform strategies to promote students’ engagement through distance-learning, mitigating negative effects of the situation.

Most universities were closed due to COVID-19 pandemic from spring 2020 to spring 2021, covering three semesters of university studies. These manifold containment measures caused university students facing a fundamentally altered situation with respect to their lives as a whole, including studies. Strict measures, such as lockdowns, restrictions on movement, disruption of routines, physical distancing, curtailment of social interactions and deprivation of traditional learning methods have taken place due to the pandemic in order to prevent the virus from spreading. COVID-19 has created unique challenges for psychological well-being, leading to increased stress, anxiety, and mental health concerns among learners worldwide (UNESCO, 2020). Thus, to counteract further negative developmental outcomes among students worldwide, it would be important to identify resources that foster resilience in times of crisis. Therefore, the present research seeks to identify how students’ study related wellbeing in terms of study burnout and engagement has changed between May 2020 and April 2021, and to identify various resources which support study engagement and prevent burnout among students in higher education institutions in this unprecedented situation. Both the demands-resources model (Salmela-Aro et al., 2021) and self-determination theory (Deci & Ryan, 2000) are used as frameworks to examine students’ engagement and burnout during the COVID-19 pandemic.

## Study Engagement and Burnout

Study burnout is a study-related syndrome including exhaustion, negative cynical attitude towards studying and feelings of inadequacy as a student (Salmela-Aro et al., 2009; Salmela-Aro & Read, 2017). Study engagement, in turn, can be defined as vigor, dedication and absorption towards studying (Salmela-Aro & Upadyaya, 2012; Salmela-Aro & Read, 2017). Recent studies have revealed that study burnout can lead to depressive symptoms and increase the risk of dropping out from studying four times more, whereas study engagement can promote both life satisfaction and success in future educational transitions (Bask & Salmela-Aro, 2013; Salmela-Aro et al., 2021; Upadyaya & Salmela-Aro, 2015). Both study engagement and burnout are multi-dimensional constructs, which consist of behavioral, affective, and cognitive components (Fredricks et al., 2004; Salmela-Aro et al., 2009; Upadyaya & Salmela-Aro, 2013), and involve dynamic and reciprocal processes between the sub-components which influence and are influenced by the study environment (Wang & Degol, 2014). Recent studies have recommended that study engagement and burnout should be investigated together (Salmela-Aro & Read, 2017; Tuominen-Soini & Salmela-Aro, 2014), and it has been suggested that study engagement and burnout provide an adequate overview of students’ study-related well-being (Salmela-Aro, 2017; Upadyaya & Salmela-Aro, 2013). Engaged students have energy and are willing to put effort into their work (i.e., vigor), they feel driven, enthusiastic, and inspired (i.e., dedication), as well as entirely concentrated in studies that they enjoy doing (i.e., absorption). In contrast, school burnout occurs when students feel overwhelmed by the demands of their studies (Schaufeli et al., 2002). As a result, these students tend to feel exhausted and pressured by their studies, may become cynical toward their studies, and come to feel inadequate or unable to reach their study goals. Study environment provides the opportunities and resources for engagement as well as demands for burnout to occur, and students’ skills, needs and motivation determine how they engage within those opportunities. Recently, understanding both the opportunities and resources that support students’ engagement and prevent from stress and burnout have become an important priority for educational policy and practice (Wang & Eccles, 2012).

## Demands -Resources Model

According to the study demands-resources model (Salmela-Aro et al., 2021), the more study-related demands, such as pressure and workload the students experience, the more study burnout they experience, whereas study-related resources, such as support from peers and teachers, often lead to study engagement. We expect that the more distance study related resources, such as teacher online support and digital support, students experience, the more likely the students are engaged (Salmela-Aro et al., 2021). In turn, study burnout can be approached as a mismatch between distance online study resources and the demands imposed by the distance study context, such as technical challenges, which cause students to experience depletion of energy without gaining appropriate returns. In line with the study demands-resources model, two processes can be identified concerning the distance study context: a motivational process, in which distance study-related resources can lead to increased study engagement, and a health impairment process in which distance study-related demands lead to study-related strain and stress and problems with mental health (see also Bakker & Demerouti, 2006). In support of the model, findings from longitudinal research have shown that study burnout and engagement also spill over from the study domain-specific context to general ill- and wellbeing (Salmela-Aro et al., 2021; Upadyaya & Salmela-Aro, 2017): engagement predicts later life satisfaction, whereas burnout predicts subsequent depressive symptoms. Longitudinal studies have shown that engagement predicts higher grades, successful educational transitions, and later satisfaction with chosen educational pathways (Upadyaya & Salmela-Aro, 2015). Study burnout, in turn, predicts decreases in educational aspirations (Salmela*-*Aro, & Upadyaya, 2017; Widlund et al., 2021), a fourfold greater likelihood of dropping out (Bask & Salmela-Aro, 2013; Parker et al., 2015), and decreases in academic achievement (Madigan & Curran, 2020). In the current study, we examine the role of distance study-related demands and resources in explaining study burnout and engagement during the COVID-19 pandemic.

Overall, the situation caused by the pandemic concerning both social distancing and distance learning has caused stress to students by requiring them to adapt quickly to novel digitally mediated study practices and to switch to social media platforms to maintain relationships with teachers and other students as well as family, and friends. While today’s students are generally well-prepared to engage with digital technologies in their studies, smaller groups of students can be identified to whom it is not the case and who are particularly vulnerable (Nguyen et al., 2020). Furthermore, less digitally adept students may experience a double burden, caused by higher digital demands due to distance learning as well as unfavorable, stress-related emotions and loneliness (Händel et al., 2020). However, students that were already well-versed with digital technologies and social media before the pandemic may have been better equipped to harvest the benefits provided by these digital tools as resources and avenues to keep up sense of relatedness (Cantanero et al., 2021), and to mitigate the negative effects of social distancing (Orben et al, 2020).

## Self-Determination Theory

In turn, according to the self-determination theory (SDT; Deci & Ryan, 2000) the three basic psychological needs for competence, autonomy, and relatedness are associated with both study-related and more general psychological well-being. Basic psychological need satisfaction can also act as a buffer in times of crisis, such as during COVID-19, reducing possible stress and promoting adaptive coping with the COVID-19 pandemic (i.e., Vansteenkiste & Ryan, 2013). Competence refers to achieving one’s goals and experiencing one’s related behavior as effective and competent. University students feel effective and competent when they are able to meet the requirements of their studies, such as progress with their courses. Autonomy refers to experiencing one’s behavior as volitional. University students feel autonomous when they willingly put effort and time to their university studies and feel internal motivation. Relatedness refers to feeling connected with other students, and experiencing mutual support from others, such as teachers, peers and parents (Deci & Ryan, 2000; Niemiec & Ryan, 2009): The need for social relatedness refers to feeling connected to and accepted by others. In the current study, we examine the role of psychological needs, competence, autonomy, and relatedness for study engagement and burnout during COVID-19.

## Demands-Resources, and Basic Psychological Needs in Times of COVID-19 Distance Education

In the context of OECD Education 2030 Learning Compass, the role of higher education institutions is not only to impart knowledge, but to develop the whole student by providing opportunities for wellbeing, thriving, and personal growth. OECD Learning Compass highlights three issues: First, the learning compass points towards wellbeing highlighting the importance of student wellbeing for learning. Second, the learning compass highlights the importance of new transformational competences, creating new value, reconciling with challenges, and taking responsibility which are in line with psychological needs of competence, autonomy, and relatedness. Third, the learning compass highlights both student agency and co-agency with peers for learning and wellbeing. Universities foster a range of transversal skills such as complex and autonomous thinking (European Commission, 2017), and enable social contacts and interaction, offer chances to build networks, establish new friendships, and provide experiences of belonging to the institution (Tonon, 2020). Universities represent an important developmental context for young people to acquire their developmental tasks and unfold their potentials, and to experience relatedness and sense of belonging in the peer group. The lockdown of universities due to the COVID-19 crisis is thus an unprecedented challenge for students’ engagement and may hinder students’ possibilities for thriving.

Rapid transitions from face-to-face classes to online distance learning environments occurred at the beginning of the pandemic when universities worldwide switched to distance education in order to enable young people to continue their studies (Marinoni et al., 2020; Murphy, 2020). Distance education refers to an umbrella term, and its implementation varies greatly, even oftentimes it involves three factors: (1) lack of physical presence, (2) less informal discourse, and (3) declines in spontaneous social interaction between students and teachers. These factors can lead to communication gaps between teachers and students, as well as gaps in understanding, which may further manifest as negative emotions, and misconceptions (Moore, 1993). However, less is known about students’ psychological needs and challenges in distance learning environments, and there is a need to examine them further. Numerous studies have shown that social relatedness is often associated with academic success in both in-person and distance learning environments, thus, it would be important to support interaction among students in any learning settings (Smith & Naylor, 2001; Tomás-Miquel et al., 2016). Moreover, consistent evidence exists showing that relatedness is an important factor contributing to one’s psychological well-being (e.g., Olsson et al., 2013; Reis et al., 2000), which further highlights the relevance of maintaining social contacts even during the pandemic, either with other students participating the same distance learning environment or with other social contacts outside of the university.

Regardless of the above mentioned challenges, distance education also bears a potential to promote students’ experiences of competence and autonomy as it provides students with opportunities to practice and study at their own pace (Paechter & Maier, 2010). Students can be challenged according to their abilities in distance education and expand them autonomously. Previous studies have revealed that autonomous, individualized learning environments create optimal learning conditions for students to experience themselves as competent (Niemiec & Ryan, 2009). According to self-determination theory, both autonomy and competence are necessary conditions for intrinsic motivation (Ryan & Deci, 2000). Moreover, relatedness has been found to be related to positive emotion, in line with previous studies pointing to the high relevance of social contacts for students’ well-being (Damon et al., 2003; Kern et al., 2015). One central issue during the COVID-19 has been physical separation between educators and learners as well as among peers, leading to limited spontaneous interaction and less informal, personal exchange.

## The Present Research

In order to support study engagement and prevent from study burnout among university students during the COVID-19 pandemic, it is important to identify various resources of study engagement and burnout which are crucial for students’ success in the current unprecedented situation.

Thus, the present study aims to examine how various demands and resources related to distance learning on the one hand, and how the three psychological needs (e.g., competence, autonomy, and relatedness) on the other hand are associated with study engagement and burnout among university students during COVID-19. In this respect, the results are analyzed using two frameworks: study demands-resources model (Salmela-Aro et al., 2021) and the SDT model (Deci & Ryan, 2000). Educational context is an important setting which provides students opportunities for personal growth and thriving, thus, the present study examines the extent to which distance study demands and resources on the one hand, and the satisfaction of basic psychological needs on the other hand, are related to university students’ study engagement and burnout during COVID-19. The associations between basic psychological need satisfaction of competence, autonomy, relatedness, and study engagement and burnout, as well as the associations between distance study demands and resources and study engagement and burnout are investigated. The specific research questions are as follows:

Our first research question (RQ1) was to investigate the changes in the level of study burnout and engagement from the beginning of the pandemic (May 2020) to two consecutive semesters (December 2020 and April 2021). We expect that study burnout increases and engagement decreases continuously across the measurement times from May 2020 to April 2021.

Our second research question (RQ2) was to investigate how distance learning demands and resources were related to study engagement and burnout, and whether these relations differ as a function of time. Based on the study demands-resources model, we expect that distance learning demands predict study burnout and resources predict study engagement, whereas the differences across different timepoints were addressed more exploratively.

The third research question (RQ3) was directed to examine how basic psychological needs were related to study engagement and burnout and whether there would be differences across timepoints. Based on the SDT, we expect that competence, autonomy, and relatedness positively predict study engagement and negatively predict burnout, and the differences across timepoints were approached more exploratively.

Finally, as the fourth research question (RQ4), we compare the extent to which the components of the two frameworks (e.g., demands-resources and psychological needs) are able to explain the variation in study burnout and engagement at different phases of the COVID-19 pandemic (e.g., May 2020, December 2020 and April 2021). However, as this is the first study to compare these two theories, demands-resources and SDT, for study engagement and burnout, we do not set any precise hypotheses.

## Method

### Participants and Procedure

#### Finnish Higher Education

The target group of this study consisted of university students, specifically in Finland. In Finland, students apply for higher education after completing upper secondary education. Universities offer a bachelor's or master's degree as well as postgraduate degrees. The target completion time for a bachelor's degree is 3 years. After graduating as a bachelor, students will continue to a master's degree with a target completion time of 2 years. A university degree is free of tuition charges in Finland and students receive financial aid from the state. On March 18th, 2020 universities stopped providing onsite learning due to COVID-19 in Finland. However, universities ensured continued education by providing distance learning.

Due to practical reasons, the data utilized in this present study consisted of three convenience samples obtained from the students of the University of Helsinki in different phases of the COVID-19 pandemic while all teaching was provided at distance in Finnish universities (April 2020, December 2020, and April 2021). Although using a convenience sample limits the generalizability of our findings, such approach allowed us to collect data after transforming to distance education. Severity of the COVID-19 situation and strictness of the restrictions were the highest in the metropolitan area, where the University of Helsinki is located. The University of Helsinki did not provide onsite teaching other than in some restricted subjects during the entire period of data collection. The overall sample comprised 4,712 students (19% males, 79% females, 2% diverse) with a mean age of 27.56 years (*SD* = 8.21). Data were collected via online questionnaires. Before answering to the items, participants were informed about the research goals, approximate duration of the questionnaire, inclusion criteria for participation, i.e., attending university, and the complete anonymity of the data. All students participated voluntarily and only those who gave active consent were included in the dataset. The link to the online questionnaire was distributed via faculty’s e-mail lists and the University of Helsinki’s social media channels. The ethical standards outlined in the Declaration of Helsinki were followed.

The May 2020 sample comprised 1,501 university students (20% males, 78% females, 2% diverse) whose mean age was 28.55 years (*SD* = 9.08, *Mdn* = 25.00, *Range* = 18–82). The December 2020 sample comprised 1,526 university students (17% males, 81% females, 2% diverse) whose mean age was 26.64 years (*SD* = 7.79, *Mdn* = 24.00, *Range* = 18–71). The April 2021 sample comprised 1,685 university students (20% males, 78% females, 2% diverse) whose mean age was 27.52 years (*SD* = 7.66, *Mdn* = 25.00, *Range* = 17–72). The data represents the students of the University of Helsinki well. There were respondents from all faculties and comprehensively from students at different stages of their studies. The most significant deviation was that the proportion of women was slightly higher (+ 11%) in the present study than what is the actual the population of the University of Helsinki, where 65% of undergraduate students are female (University of Helsinki, 2020).

## Measures

*Study Burnout* was examined with the Study Burnout Inventory (Salmela-Aro & Read, 2017; Salmela-Aro et al., 2009) consisting of ten items measuring study burnout: (1) feelings of exhaustion when studying (e.g., *“I feel overwhelmed by my studies”*); (2) cynicism towards the meaning of studying (e.g., *“I feel that I am losing interest in my studies”*), and (3) sense of inadequacy at studies (i.e., *“I often have feelings of inadequacy in my studies”*). The responses were rated on a 6-point scale (1 = strongly disagree; 6 = strongly agree). The Cronbach’s alpha for the study burnout scale was 0.94, which shows excellent reliability.

*Study engagement* was measured using study engagement inventory (Salmela-Aro & Read, 2017; Salmela-Aro & Upadyaya, 2012). The scale consisted of nine items measuring experiences of energy (i.e., *“When studying I am bursting with energy”*), dedication (i.e., *“I find the studying full of meaning and purpose”* and *“I am enthusiastic about my studies”*), and absorption (i.e.,*”Time flies when I’m studying”*). The responses were rated on a 6-point scale (1 = strongly disagree; 6 = strongly agree). The Cronbach’s alpha for the study engagement scale was 0.91 showing strong reliability.

*Competence* was measured with three items adapted from the Work-related Basic Need Satisfaction Scale (W-BNS; Van den Broeck et al., 2010). We adapted the work-related items to the university context (sample item: “Currently, I am dealing well with the demands of my studies”). The Cronbach’s alpha was 0.88 meaning reliability was good.

*Autonomy* was assessed with two newly developed items that addressed the extent to which students felt that they were self-determined in approaching their studies in the current situation (sample item: “Currently, I can perform tasks in the way that best suits me”). The Cronbach’s alpha for autonomy was 0.76 showing good reliability.

*Relatedness* was measured with three items inspired by the Work-related Basic Need Satisfaction Scale (W-BNS; Van den Broeck et al., 2010) and the Basic Psychological Need Satisfaction and Frustration Scale (Heissel et al., 2018). In contrast to competence and autonomy, the items targeting relatedness did not solely refer to the university context, but also to significant others in general (sample item: “Currently, I feel connected with the people who are important to me”). The Cronbach’s alpha was 0.76, which shows good reliability.

*Distance Study Demands* included five questions related the key demands during distance studying as follows: “At the current situation it is very demanding: 1. To plan my daily schedule, 2. That I have to take care of so many things, 3. Technical challenges (such as slow wi-fi, problems with the computer), 4. I do not have a place where I can study without interruptions, and 5. Many issues disturb my studying” to be rated from 1 (totally disagree) to 5 (totally agree). The Cronbach’s alpha for distance study demands was 0.73 showing good reliability.

*Distance Study Resources* included four questions related to the key resources during distance studying as follows: “Please evaluate the following resources: 1. I can study well in the digital context. 2. Most of the teachers know well how to teach in the digital context. 3. I get good support from teachers at the current distance context. 4. Most of the teachers are often connecting with the students” to be evaluated between 1 (totally disagree) and 5 (totally agree). The Cronbach’s alpha for distance studying was 0.77 showing good reliability.

*Background.* The following background variables were gathered: Age, gender, the beginning year of studies, study field, working situation, and number of children.

## Data Analysis

As preliminary analyses we investigated the data for outliers and missing data, and the code for data wrangling as well as additional materials can be downloaded from https://osf.io/6d35r/. The research questions were analyzed using linear mixed models with study engagement and study burnout as dependent variables, respectively, and measurement time (with Time 1 as baseline reference group), basic psychological needs, and study demands and resources as independent variables. To control for the non-independence of data points due to participants being clustered within faculties and starting years, the tenability of the random intercepts (by faculty*year) were decided by likelihood ratio tests (e.g. Harrison et al., 2018).

More precisely, to answer RQ1, that is, to examine change in study engagement and burnout over time, we specified models (Models M1a and M1b) with measurement time as the sole predictor. In these models, Times 2 and 3 were included as dummy-coded predictors, and Time 1 was included as the baseline reference group. In M1a and M1b the intercept should be interpreted as the baseline mean for the dependent variable, whereas positive or negative Times 2 or 3 effects would indicate cumulative change over time (in comparison to the baseline). To answer RQ2, distance study related demands and resources alongside with their interaction terms with measurement time were included as predictors of study engagement and burnout (Models M2a and M2b). To answer RQ3, basic psychological needs and their interaction terms with measurement time were included as predictors of study engagement and burnout (Models M3a and M3b). The direct effects from demands, resources, and psychological needs to engagement and burnout in M2a to M3b should be interpreted as the effects of the predictor at baseline (Time 1), whereas the Time*Predictor interaction terms differing from zero would indicate that the effect of the predictors is higher/lower in certain timepoint in comparison to baseline, which further indicates change over time in the dependent variables. In order to examine RQ4 further, we compared the two theories by estimating the models 2 and 3 separately for each measurement point and compared how well the two theories explained variance in study engagement and burnout by using marginal R^2^ with bootstrapped 95% confidence intervals—non-overlapping intervals indicating differing explanatory power.

## Results

Table 1 shows the descriptive values and Cronbach’s alphas for the combined data, the values for separate timepoints are presented in additional material. Only 2.9% of the data was missing, and the missingness occurred completely at random (Little’s MCAR test chi-square (3767) = 1467, *p* = 1).

**Table 1:** Summary of descriptive values

	Mean	SD	Cronbach's Alpha
Study engagement	2.95	1.14	0.91
Study burnout	3.27	1.31	0.94
Distance study demands	3.13	0.90	0.73
Distance study resources	3.20	0.88	0.77
Autonomy	3.28	1.04	0.76
Competence	3.44	1.02	0.88
Relatedness	3.10	0.95	0.76

**Fig. 1:**
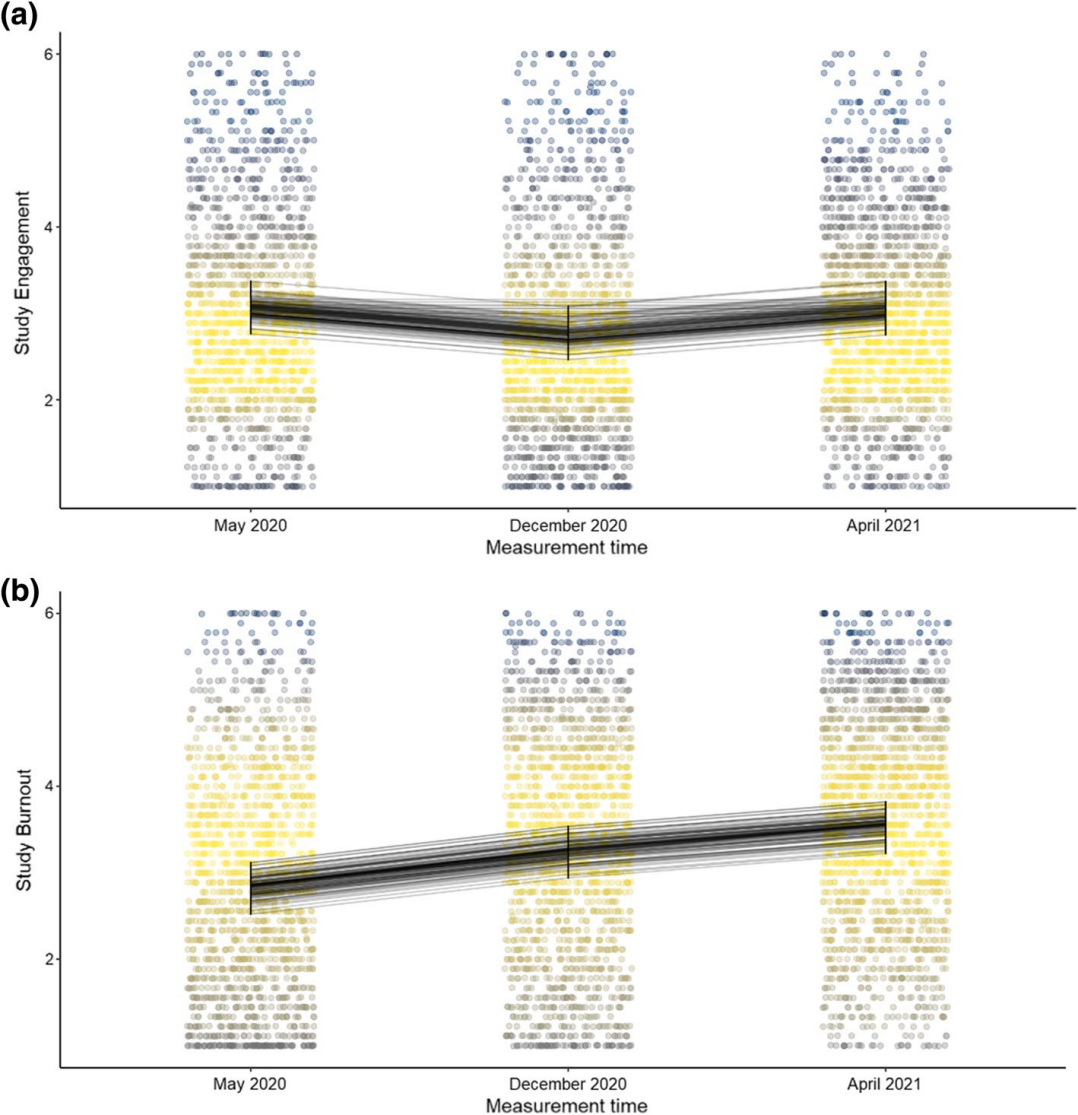
**a** Study engagement as a function of time. **b** Study burnout as a function of time. Note: the small lines in the figure indicate the random intercept

Regarding the RQ1, the results for M1a showed that the Time 2 effect (see Table 2) was negative and differed from zero indicating that study engagement (see Fig. 1a) was lower in December 2020, whereas it appeared to bounce back during April 2021 (when the Time 3 effect was not different from zero). In turn, the results for M1b showed that both Time 2 and Time 3 effects were positive and differed from zero (see Fig. 1b and Table 2), which indicated that study burnout increased linearly across the measurements.

**Table 2:** Study engagement and study burnout as a function of time

Predictors	Study Engagement			Study Burnout		
	Estimates	CI	*p*	Estimates	CI	*p*
(Intercept) [Spring 2020]	3.06	2.99–3.13	** < 0.001**	2.83	2.76–2.91	** < 0.001**
Time 2 [December 2020]	− 0.30	− 0.38 to − 0.22	** < 0.001**	0.42	0.33–0.51	** < 0.001**
Time 3 [April 2021]	− 0.01	− 0.09–0.07	0.753	0.70	0.61–0.79	** < 0.001**

*Note*: Time 2 and Time 3 are dummy-coded with Time 1 as reference group mean of which is captured by the intercept which is random by faculty*starting year, p-values added using Satterthwaite's degrees of freedom method via lmerTest (Kuznetsova et al., 2017)

Regarding RQ2 (see Table 3 for parameter estimates), the results for M2a indicated that while the Time 1 distance study demands were negatively related to study engagement, the Time 2*Distance study demands interaction effect did not differ from zero, showing no difference between Time 1 and Time 2, whereas the Time 3*Distance study demands interaction effect was positive, indicating that negative effect of study demands on study engagement was smaller in April 2021 than in previous measurements. In turn, the positive effect of distance study resources on study engagement remained stable over time (the Time 2 and Time 3 interaction effects did not differ from zero). Regarding study burnout, the pattern was similar. The results for M2b showed that demands were positively related to study burnout at Time 1, the Time 2*Distance study demands interaction was not different from zero, however, the significant Time 3*Distance study demands interaction indicated that the positive effect of study demands on study burnout was slightly lower in April 2021. In turn, study resources were negatively related to burnout at Time 1, and the Time 2* Distance study resources interaction was not different from zero, however, the significant Time 3*Distance study resources interaction indicated that the negative effect of study resources on study burnout was slightly lower in April 2021 than in previous measurement times.

**Table 3:** Distance study demands and resources as predictors of study engagement and study burnout

Predictors	Study engagement			Study burnout		
	Estimates	CI	*p*	Estimates	CI	*p*
(Intercept)	2.19	1.88–2.51	** < 0.001**	2.57	2.26–2.88	** < 0.001**
Time 2 [December 2020]	− 0.17	− 0.61–0.27	0.458	0.35	− 0.09–0.79	0.118
Time 3 [April 2021]	− 0.10	− 0.53–0.32	0.636	0.51	0.09–0.93	**0.017**
Distance study demands [Time 1]	− 0.31	− 0.37 to − 0.26	** < 0.001**	0.68	0.62–0.73	** < 0.001**
Distance study resources [Time 1]	0.53	0.47–0.59	** < 0.001**	-0.54	− 0.60 to 0.48	** < 0.001**
Time 2*Distance study demands	− 0.06	− 0.14–0.02	0.144	0.02	− 0.06–0.10	0.651
Time 3*Distance study demands	0.12	0.04–0.20	** < 0.001**	-0.09	− 0.17 to − 0.02	**0.019**
Time 2*Distance study resources	0.08	− 0.01–0.16	0.079	-0.05	− 0.14–0.03	0.224
Time 3*Distance study resources	− 0.03	− 0.11–0.05	0.487	0.09	0.00–0.17	**0.038**

Time 2 and Time 3 are dummy-coded with Time 1 as reference group, intercept represents the value of the dependent variable in the hypothetical case of having all predictors at zero and is random by faculty*starting year, p-values added using Satterthwaite's degrees of freedom method via lmerTest (Kuznetsova et al., 2017)

Regarding RQ3 (see Table 4 for parameter estimates), the results for M3a indicated that sense of autonomy and competence were related to higher study engagement in Time 1, whereas sense of relatedness showed no effect. The effect of autonomy did not increase over time (Time 2*Autonomy and Time 3*Autonomy interactions did not differ from zero), but there was a positive Time 2*Competence interaction, indicating that the effect of competence on study engagement was greater in December 2020 compared to May 2020. Regarding sense of relatedness, in turn, the Time 2*Relatedness interaction was not different from zero but the Time 3*Relatedness interaction was, indicating that sense of relatedness became a positive predictor of study engagement only in April 2021. Regarding study burnout, both sense of autonomy and competence were negatively related to study burnout, showing no change over time (Times 2 and 3 interactions did not differ from zero). However, relatedness was positively, yet the estimate was very close to zero, related to study burnout in Time 1, but the Time 2*Relatedness and Time 3*Relatedness interactions were both negative indicating that sense of relatedness became a negative predictor of study burnout in December 2020 and April 2021.

**Table 4:** Basic psychological needs as predictors of study engagement and study burnout

Predictors	Study engagement			Study burnout		
	Estimates	CI	*p*	Estimates	CI	*p*
(Intercept)	0.69	0.45–0.93	** < 0.001**	6.03	5.79–6.27	** < 0.001**
Time 2 [December 2020]	− 0.56	− 0.88 to − 0.23	**0.001**	0.68	0.36–1.00	** < 0.001**
Time 3 [April 2021]	− 0.11	− 0.42–0.20	0.481	0.74	0.44–1.05	** < 0.001**
Autonomy [Time 1]	0.19	0.14–0.25	** < 0.001**	− 0.28	− 0.34 to − 0.23	** < 0.001**
Competence [Time 1]	0.45	0.39–0.50	** < 0.001**	− 0.67	− 0.73 to − 0.62	** < 0.001**
Relatedness [Time 1]	0.02	− 0.04–0.08	0.500	0.06	0.01–0.12	**0.028**
Time 2 *Autonomy	− 0.05	− 0.12–0.03	0.216	− 0.00	− 0.08–0.07	0.937
Time 3 *Autonomy	− 0.04	− 0.11–0.04	0.316	0.03	− 0.04–0.11	0.396
Time 2 *Competence	0.12	0.04–0.20	** < 0.001**	− 0.02	− 0.10–0.06	0.577
Time 3 *Competence	0.05	− 0.02–0.13	0.181	− 0.02	− 0.10–0.06	0.634
Time 2 *Relatedness	0.06	− 0.02–0.13	0.161	− 0.12	− 0.20 to − 0.04	**0.002**
Time 3 *Relatedness	0.11	0.03–0.18	**0.007**	− 0.14	− 0.22 to − 0.07	** < 0.001**

Time 2 and Time 3 are dummy-coded with Time 1 as reference group, intercept represents the value of the dependent variable in the hypothetical case of having all predictors at zero and is random by faculty*starting year, p-values added using Satterthwaite's degrees of freedom method via lmerTest (Kuznetsova et al., 2017)

**Fig. 2:**
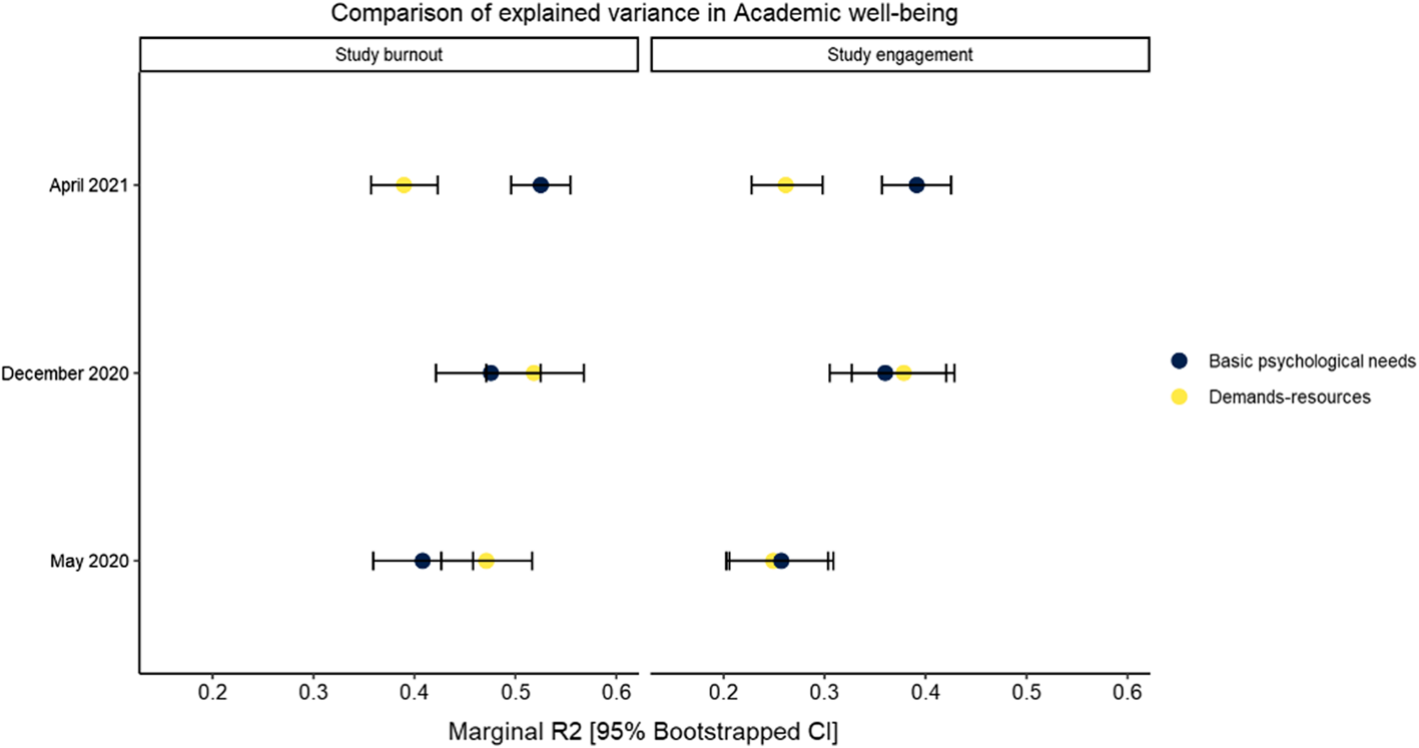
Comparison of R^2^ for the two theories across the timepoints

Regarding RQ4 (see Fig. 2 and Table 5) the marginal variance explained (R^2^) in study engagement and burnout scores by both demands-resources and basic psychological needs increased for December 2020. However, in April 2021 the demands-resources model explained considerably less variance than in 2020 compared to basic psychological needs, as indicated by the non-overlapping bootstrapped 95% confidence intervals. Moreover, the non-overlapping confidence intervals in April 2021 between the two modes indicate that demands-resources explained considerably less variance than basic psychological needs satisfaction. In turn, the explanatory power of basic psychological needs seemed to increase linearly.

**Table 5:** Comparison of variance explained in each time point

Model	May 2020			December 2020			April 2021		
	R^2^	95%	C.I	R^2^	95%	C.I	R^2^	95%	C.I
*Study engagement*									
Demands-resources	0.25	0.20	0.30	0.38	0.32	0.44	0.26	0.22	0.30
Basic psychological needs	0.26	0.21	0.31	0.36	0.31	0.42	0.39	0.36	0.42
*Study burnout*									
Demands-resources	0.47	0.43	0.52	0.52	0.47	0.57	0.39	0.36	0.42
Basic psychological needs	0.41	0.36	0.46	0.48	0.42	0.53	0.53	0.50	0.55

*Note*: R^2^: Marginal R2 (variance explained) for models run separately in each timepoint, 95% C.I.: confidence interval obtained via 1000 bootstrap draws

## Discussion

Due to the COVID-19 pandemic many universities closed their doors from in-person teaching for over one and a half years. These changes likely had severe implications on university students’ study burnout and engagement. The present study examined how study-related burnout and engagement developed among students across the COVID-19 pandemic in University of Helsinki. In addition, the associations between distance study demands and resources, psychological needs, and study burnout and engagement were examined. Supporting our expectations, the results showed that study burnout increased across the time points, being the highest in April 2021, whereas study engagement was the lowest in December fall 2020. However, study engagement bounced back during the second spring (2021) of the pandemic to the same level with the first COVID-19 spring (May 2020). Supporting our expectations, distance study-related demands were associated with lower study engagement and higher burnout, whereas resources were associated with higher study engagement and lower burnout, and the role of distance study demands attenuated during April 2021. It is possible that at the beginning of the pandemic when University of Helsinki students were still getting used to the life under the new restrictions caused by the pandemic, they also faced more distance study demands (e.g., planning daily schedules, technical challenges). However, as time passed on, students acquired new skills on how to manage their daily lives and distance study challenges, and the role of such demands in their study burnout reduced, and the role of psychological needs, such as the need for relatedness, became more pronounced.

Supporting our expectations related to RQ3, the results showed that while generally the influence of autonomy and competence in predicting higher study engagement and lower burnout were strong in May 2020, the role of relatedness seemed to increase during the pandemic—especially concerning study burnout. The results showed that especially in April 2021 relatedness was negatively associated with study burnout. This result is likely to reflect the long period of isolation and increased loneliness among the university students during the long lockdown period. Thus students’ social contacts reduced drastically, increasing students’ stress, loneliness (Zheng et al., 2021), and burnout (Salmela-Aro et al., 2021), and when the situation became prolonged, our results showed that the effect of relatedness became more pronounced and an important factor reducing burnout. In addition, it is possible that over the pandemic, students learned new ways how to strengthen their connections to friends and peers, which manifested as relatedness in the present study, and was further negatively associated with burnout symptoms. For example, university of Helsinki provided increasing support for students across the pandemic (e.g., online resources and support from psychologists, instructions and tips for remote studying, support and online courses to promote personal well-being), which might have increased students’ feelings of relatedness at the later phases of the pandemic.

When comparing the role of basic psychological need satisfaction (experienced competence, autonomy, and relatedness) and distance study-related demands (technical challenges, planning daily schedules) and resources (ability to study in digital context, support from teachers in distance learning context) with study engagement and burnout, it was found that at the beginning of the pandemic the explanatory power of demands and resources was strong. However, later on during the pandemic the role of psychological needs increased, supporting our expectations. The explanatory power of both demands-resources and basic psychological needs increased during December 2020 whereas in April 2021 the demands-resources model explained considerably less of the variation in burnout and engagement compared to December 2020, and compared to the concurrent basic psychological needs satisfaction. In turn, the explanatory power of basic psychological needs seemed to increase linearly during the pandemic.

## Implications for University Studies in Times of Crises

While limited in scope to Finnish students from a single university, these results can be utilized to inform strategies to promote students’ well-being through distance-learning, mitigating the possible negative effects of such conditions in current and future crises, as well as to inform future studies. For example, at the beginning of a crisis situation students may need more support concerning coping with distance study demands (e.g., technological support, scheduling support), whereas later on, if the crisis is prolonged, support related to basic psychological needs (e.g., social support, promoting sense of belonging) would be required. This was indicated by the findings showing that elements of self-determination theory, in terms of basic psychological needs, explained over 50% (R^2^ = 0.53) of the variation in study burnout during April 2021. Accordingly, the present study identified a high relevance of competence, autonomy, and relatedness in later phases of COVID-19 for university of Helsinki students’ study engagement and burnout. Even in a distance-learning context, it is possible to promote all of the three basic psychological needs among university students. Based on the identified high relevance of students’ competence, distance education in times of crisis is required to explicitly enable students to experience successes. Through individualized, personalized, and simultaneously autonomy-supportive learning opportunities it would be possible to provide experiences of success. For example, by challenging students based on their individual resources and strengths. Experiences of success can be also promoted by setting intermediate goals and enough individual feedback (Oliveira et al., 2018). University students miss individual discussion concerning their studies, and thus individual, personalized support and guidance from the university staff and teachers to students could be provided via online digital platforms. Moreover, promoting relatedness is highly important, and identification with the university in the current situation, and digital learning platforms can be used to enable online group work in a situation where physical distance is required. To foster feelings of learning together as a group, synchronous learning units should be used to reflect on learning processes, successes, as well as struggles and to promote cohesion within the group. Moreover, in crisis situations universities should make every effort to reopen as soon as possible after the closures, as public health restrictions allow.

## Limitations and Future Directions

As a limitation it is important to underline that due to practical reason the samples were obtained as convenience samples which limits the generalizability both to Finnish students in general as well as internationally. In addition, the data collection in each sample was cross-sectional by nature, and modelled as such, yet we cannot be certain whether some students participated more than once. The inclusion of the random intercept at the faculty and starting year level, however, adjusts for this uncertainty to a certain extent. University of Helsinki student population were well represented, with the exception that female students were overrepresented in the sample (+ 11%). Such gender imbalance is likely to induce some bias as female students tend to report both higher study engagement and study burnout. In addition, we cannot separate random sampling variation from variation caused by the continuing pandemic situation and context—that said, the effects found are, however, theoretically predictable.

## Data Availability

We will include the data osf.io. All materials needed to reproduce the analysis can be downloaded from: https://osf.io/6d35r/.

## Code availability

We will include the codes according to osf. As preliminary analyses we investigated the data for outliers and missing data, the code the follow the data wrangling as well as additional material can be downloaded from osf-link. All materials needed to reproduce the analysis can be downloaded from: https://osf.io/6d35r/.
